# A retinal imaging system for combined measurement of optic nerve head vascular pulsation and stimulated vasodilation in humans

**DOI:** 10.1038/s41598-023-44390-2

**Published:** 2023-10-10

**Authors:** Anthony E. Felder, Mansour Rahimi, Amir Nankali, Nathanael Matei, Farzan Abdolahi, Norman P. Blair, Mahnaz Shahidi

**Affiliations:** 1 Richard and Loan Hill Department of Biomedical Engineering, University of Illinois at Chicago, Chicago, IL USA; 2 Department of Ophthalmology, University of Southern California, Los Angeles, CA 90033 USA; 3 Department Ophthalmology and Visual Sciences, University of Illinois at Chicago, Chicago, IL 60607 USA

**Keywords:** Retinal diseases, Biomedical engineering, Imaging and sensing

## Abstract

Vascular pulsation at the optic nerve head (ONH) reflects vessel properties. Reduction in the stimulated retinal vasodilatory capacity has been reported in diabetes, but its relation with vascular pulsation is unknown. Here we report a new retinal imaging system for correlative assessment of ONH vascular pulsation and stimulated retinal vasodilation. Retinal reflectance images were acquired before and during light flicker stimulation to quantify arterial and venous vasodilation (D_A_R, D_V_R) in subjects with and without diabetic retinopathy (N = 25). ONH vascular pulsation amplitude and frequency (PA, PF), were quantified by curve fitting of periodic intensity waveforms acquired in retinal vasculature (RV) and ONH tissue (ONH_T_) regions. The relationships between pulsation metrics, heart rate (HR), intraocular pressure (IOP), and vasodilatory responses were evaluated. Pulsation metrics were not significantly different between regions (*p* ≥ 0.70). In RV, inter-image variabilities of PA and PF were 10% and 6%, whereas inter-observer variabilities were 7% and 2% respectively. In both regions, PF was correlated with HR (*p* ≤ 0.001). PA was associated with D_A_R in both regions (*p* ≤ 0.03), but only with D_V_R in RV (*p* ≤ 0.05). Overall, ONH vascular pulsation was associated with stimulated retinal vasodilation, suggesting diabetes may have concomitant effects on retinal vasculature compliance and neurovascular coupling.

## Introduction

Adequate blood flow is necessary for supplying oxygen and nutrients to the retinal tissue via the central retinal artery through the optic nerve head (ONH). At the ONH, the effects of transient pressure differentials both along the vessel length and across the vessel wall are thought to drive vascular pulsation, which is a periodic change in vessel diameter that occurs according to its compliance^1,2^. Thus, vascular pulsation dynamics reflect vascular structural properties and the characterization of these properties in disease, especially those affecting ocular hemodynamics, is of interest.

Vascular pulsation at the ONH has been assessed by a variety of techniques. One technique is measurement of vascular diameter over time using fundus imaging systems such as the dynamic vessel analyzer (DVA, Imedos, Jena, Germany)^3^. This system, and others like it, have been used to evaluate retinal venous pulsation at the ONH^4,5^. Another technique is laser speckle flowgraphy (LSFG)^6^, which has been used to derive average pulsation waveforms at the ONH^7,8^ and determine blood vessel stiffness, intima-media thickness, and severity of carotid arterial plaque^9,10^. Imaging photoplethysmography (IPPG) has also been used to detect pulsatile blood volume changes either within select retinal vessels or the ONH tissue^11–14^, but not comparatively within the same subjects. IPPG-based vascular pulsation metrics have been shown to be related to heart rate, respiration rate, oxygen saturation, and alterations in compliance^15,16^. Thus far, given the relation to pressure differentials, vascular pulsation dynamics have primarily been studied in glaucoma. Specifically, using the DVA, retinal venous pulsation was found to be decreased in glaucomatous eyes^17^. With LSFG, changes in ONH pulsation waveforms were reported following glaucoma treatment^18^. Moreover, IPPG was used to demonstrate the amplitude of vascular pulsation was reduced in glaucoma subjects^13^. In diabetes, assessment of ocular pulse amplitude, which is based on intraocular pressure measurement and reflects choroidal circulation, has shown variable results^19–21^. However, to the best of our knowledge, ONH pulsation metrics derived by IPPG have not been reported in diabetes.

Diabetes is known to alter retinal vasculature, hemodynamics, and neurovascular coupling^22,23^. One established method to assess neurovascular coupling in the retinal tissue is measurement of vasodilation during light flicker stimulation. Specifically, diffuse light flicker stimulation increases neural activity^24^, dilates vasculature^25^, augments oxygen delivery and metabolism^26^, and alters the oxygen extraction fraction differentially in health and diabetes^27,28^. Given diabetes is a systemic vascular disease, it may have a concomitant effect on the retinal vasculature at the ONH and in the peripapillary region, which can be evaluated by ONH vascular pulsation dynamics and vasodilatory capacity, respectively. Here, we report a new retinal imaging technique for combined assessment of ONH vascular pulsation and stimulated retinal vasodilation. This system was developed by modifying our existing retinal oximetry and stimulated vasodilation imaging system^29^ to also assess ONH pulsation using a custom IPPG-based approach. We assessed the correlation between vasodilation and ONH pulsation in subjects with and without diabetic retinopathy.

## Methods

### Subjects

This research was approved by the Institutional Review Board at the University of Southern California (approval #19-00575). Prior to enrollment, the research study was explained to subjects and informed consent was obtained. The study adhered to tenets of the Declaration of Helsinki. For imaging, subjects’ pupils were dilated, and subjects were seated at a modified slit lamp biomicroscope with head and chin rest. Subjects were light-adapted and imaging was performed with room lights turned off. Twenty-five subjects were evaluated in the current report and were classified based on clinical retinal examination into three groups: non-diabetic subjects without any ocular disease (ND; N = 7), diabetic subjects without retinopathy (NDR; N = 7), or diabetic subjects with non-proliferative retinopathy (NPDR; N = 11; 7 mild, 4 moderate). Prior to imaging, age, heart rate (HR), mean arterial pressure (MAP), intraocular pressure (IOP), and glycated hemoglobin (HbA1C) were recorded. Ocular perfusion pressure (OPP) was calculated with the following equation: OPP = 2/3*(MAP-IOP).

### Retinal imaging system

Our previously described retinal imaging system based on a modified Zeiss slit-lamp biomicroscope^29^ was modified for combined assessments of vasodilatory response and ONH pulsation. A customized computer control program was developed using LabView (LabView 2013, National Instruments, Austin, TX, USA) with an Arduino microprocessor (Arduino Duemilanove; Arduino, Ivrea, Italy) for acquiring images under two protocols. In the first protocol, retinal reflectance images were acquired both before and during light flicker stimulation for the assessment of retinal vasodilation. Light flicker stimulation is a functional challenge to the retina that increases its metabolic activity, resulting in retinal vasodilation as part of a functional hyperemic response. Reflectance image sequences centered on the ONH were acquired at 10 × magnification (~ 5 × 5 mm field of view) using light at 570 nm and an acquisition rate of 10 Hz for a duration of one second, both before and during light flicker stimulation. The light flicker stimulation was delivered at a frequency of 10 Hz for a duration of one minute, consistent with our previous studies^29^. In the second protocol, retinal reflectance images were acquired for the IPPG assessment of ONH pulsation. IPPG is an optical imaging technique that measures blood volume changes based on variations in intensity of reflected light. Reflectance image sequences centered on the ONH were acquired at 25 × magnification (~ 2 × 2 mm field of view) using light at 570 nm and an acquisition rate of 24 Hz for a duration of three seconds. The image magnification and acquisition rate for the first protocol were selected to be consistent with our previous methodology evaluating the vasodilatory effects of light flicker stimulation. The second protocol incorporated a higher image magnification to permit the collection of more pixel intensity data from the papillary region and a greater image acquisition rate to enable higher temporal resolution for data analysis.

### Retinal image analysis: vasodilation

Image sequences were analyzed using a customized algorithm developed in Matlab (Matlab 2015, MathWorks, Natick, MA, USA) to measure retinal vessel diameters as previously described^29^. First, the retinal vasculature within a circumpapillary region of interest were segmented using a Hessian-based Frangi filter. Second, using Euclidian distance transform, the centerlines of segmented vessels were determined. Third, from these centerlines, perpendicular intensity profiles were generated periodically along the vessel length orthogonal to its curvature. Fourth, the full-width at half-maximum of the perpendicular intensity profiles was used to measure vessel diameter. Diameters were averaged among vessels and then among vessel type. Retinal vessels were manually classified as artery or vein based on visual inspection of the caliber and pixel intensities by a trained observer, such that veins appeared thicker and darker. Average arterial and venous diameter (D_A_, D_V_) were measured both before and during light flicker stimulation, and the flicker-induced ratios (D_A_R, D_V_R) were calculated as the value measured during light flicker divided by the value measured before light flicker.

### Retinal image analysis: pulsation

**Figure 1:**
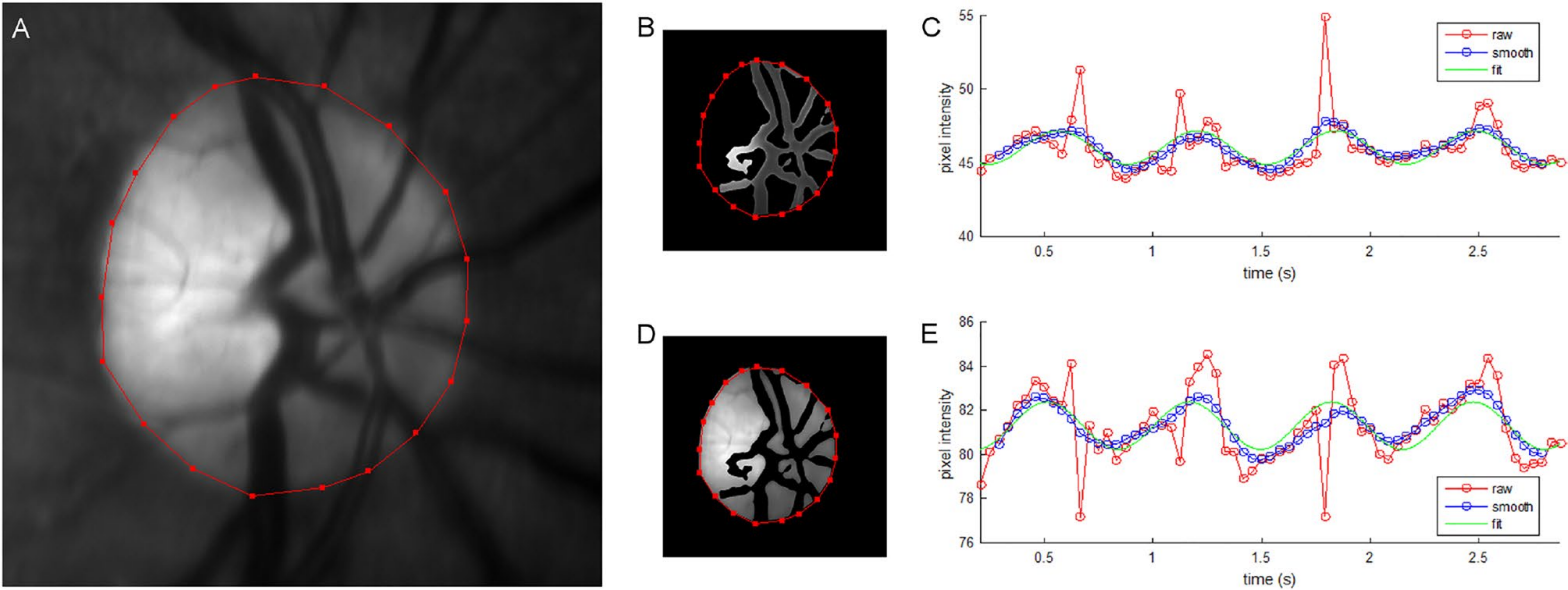
(**A**) An example of the projection image generated from a registered image sequence acquired in a non-diabetic subject. The red polygon overlay represents the border of the ONH. Regions of interest corresponding to the retinal vasculature (RV, **B**) and ONH tissue (ONH_T_, **D**) were identified. The pixel intensities from the RV (**C**) and ONH_T_ (**E**) regions of interest were averaged for each video frame and plotted as a function of time. Acquired and temporally smoothed data are denoted by red and blue circles, respectively. The fitted waveform to the data is shown in green. Spikes in the acquired data (red circles in **C**, **E**) correspond to eye motion that blurred the images during image acquisition and were removed by a smoothing filter.

IPPG image sequences were visually inspected to discard those with blinks, poor image focus, large eye movement, or reduced contrast. One image sequence per eye was selected for analysis using a customized set of algorithms developed in Matlab (Matlab 2015, MathWorks, Natick, MA, USA). First, the image sequence was automatically registered using the *normxcorr2* normalized 2-D cross-correlation and an average projection of the registered image sequence was generated. Second, to differentiate pulsation in the retinal vasculature from that of the ONH tissue, a manual polygon was used to mask the ONH and a Hessian-based Frangi filter with thresholding^30^ was used to segment the retinal vessels. While the Frangi filter has been demonstrated for vessel segmentation in a circumpapillary region^27–29^, here a trained observer manipulated vessel geometric and thresholding settings to segment only the dominant, large vasculature at the ONH. Combination of this mask and segmentation defined two regions of interest (ROI), namely, the retinal vasculature at the ONH (RV) and the ONH tissue (ONH_T_). Figure 1 shows an example of a registered projection image from a non-diabetic subject and the segmented ROIs. Third, from each ROI, a waveform was generated by plotting the average pixel intensity as a function of time.

To correct for non-pulsatile illumination changes during image acquisition, a linear regression analysis was performed on the waveform to remove changes in light intensity over time. The waveform was then smoothed with a running five-data-point averaging filter to minimize noise and a buffer of two data points at the start and end of the waveform were removed accordingly. Thus, the final number of smoothed data points for waveform fitting was 68. The smoothed time waveform (y(t)) was fitted to a general periodic function of the following form: y(t) = A*sin((B*t) + C) + D, where A, B, C, and D are coefficients of the periodic amplitude, frequency, phase, and offset, respectively. Here we define two pulsation metrics: pulsation amplitude (PA) and pulsation frequency (PF), which were the values of coefficients A and B, respectively. PF values were converted to units of beats per minute (BPM). PA and PF represent the magnitude and frequency of blood volume changes that occur during the cardiac cycle, respectively. Figure 1 shows examples of acquired, smoothed, and fitted waveforms generated from RV and ONH_T_ ROIs. Notably, in Fig. 1, the Frangi segmentation identified the dominant, large vasculature at the ONH as desired, although it also overestimated a small portion of curved vasculature to the left of the ONH center.

### Statistical analyses

Statistical analyses were performed on data from 25 subjects, after exclusion of data from three subjects based on low goodness of fitted waveforms (R^2^ < 0.23) from one or both ROIs. This R^2^ threshold corresponded to the lower 10-th percentile of R^2^ values in all subjects. Inter-image variability of pulsation metrics was calculated by percent difference of metrics obtained from two repeated image sequences in four ND subjects. Inter-observer variability was calculated by percent difference of metrics obtained by two independent observers on the same images in all non-excluded 25 subjects. One-way ANOVA was used to compare systemic/ocular metrics (age, HR, MAP, IOP, OPP, HbA1c) among disease groups (ND, NDR, NPDR). Paired t-tests were used to compare pulsation metrics (PA, PF) between ROIs (RV, ONH_T_). The relationship between metrics was evaluated by Pearson correlation analyses. All statistical analyses were performed using SPSS (v26, SPSS, Chicago, IL, USA). Statistical significance was accepted at *p* ≤ 0.05.

## Results

### Systemic and ocular metrics

Mean and standard deviation of systemic and ocular metrics stratified by disease group are provided in Table 1. Among disease groups, there was no significant difference in age, HR, MAP, IOP, or OPP (*p* ≥ 0.40). There was a significant difference in HbA1c (*p* = 0.01) among disease groups.

**Table 1:** Mean and standard deviation of systemic metrics in subjects without diabetes and at different stages of retinopathy.

	N	Age (years)	HR (BPM)	MAP (mmHg)	IOP (mmHg)	OPP (mmHg)	HbA1c (%)
All Subjects	25	60 ± 12	70.0 ± 10.7	99.9 ± 13.5	14.5 ± 2.80	52.1 ± 9.05	6.85 ± 1.46
ND	7	56 ± 14	71.0 ± 11.7	98.7 ± 7.45	13.7 ± 2.87	52.1 ± 5.49	5.50 ± 0.69
NDR	7	63 ± 15	67.3 ± 9.21	95.1 ± 10.1	15.0 ± 2.45	48.2 ± 4.40	7.30 ± 1.15
NPDR	11	60 ± 9	71.2 ± 11.5	103.7 ± 17.6	14.6 ± 2.80	54.5 ± 12.3	7.42 ± 1.51
*p*-value	–	0.55	0.74	0.42	0.69	0.40	0.01

*HR* heart rate, *BPM* beats per minute, *MAP* mean arterial pressure, *IOP* intraocular pressure, *OPP* ocular perfusion pressure, *HbA1c* hemoglobin A1c. *ND* non-diabetic subjects, *NDR* diabetic subjects without retinopathy, *NPDR* diabetic subjects with non-proliferative retinopathy.

### Pulsation and vasodilation metrics

In RV and ONH_T_, average inter-image variabilities for PA were 10% and 20%, respectively, whereas values for PF were 6% and 5%, respectively (N = 4). Average inter-observer variabilities of PA were 7% and 3% in RV and ONH_T_, respectively, whereas values for PF were 2% and 0.3% in RV and ONH_T_, respectively (N = 25). Pulsation metrics and the R^2^ values of the periodic waveform fits for both ROIs (RV and ONH_T_) are provided in Table 2. There was no significant difference in PA or PF between ROIs (*p* ≥ 0.70), but the R^2^ of fits were greater in RV than in ONH_T_ (*p* = 0.003). Mean and standard deviation of D_A_R and D_V_R in all subjects were 1.03 ± 0.09 and 1.04 ± 0.07, respectively (N = 25).

**Table 2:** Pulsation amplitude (PA) in arbitrary units (au), pulsation frequency (PF) in beats per minute (BPM), and R^2^ of periodic waveform fits to data from the retinal vasculature at the optic nerve head (RV) and in the optic nerve head tissue (ONH_T_).

	PA (au)	PF (BPM)	R^2^
RV	1.83 ± 1.15	70.8 ± 9.91	0.62 ± 0.20
ONH_T_	1.81 ± 1.25	70.4 ± 9.34	0.57 ± 0.21
*p*-value	0.94	0.69	0.003

### Relation between pulsation, systemic/ocular, and vasodilation metrics

**Figure 2:**
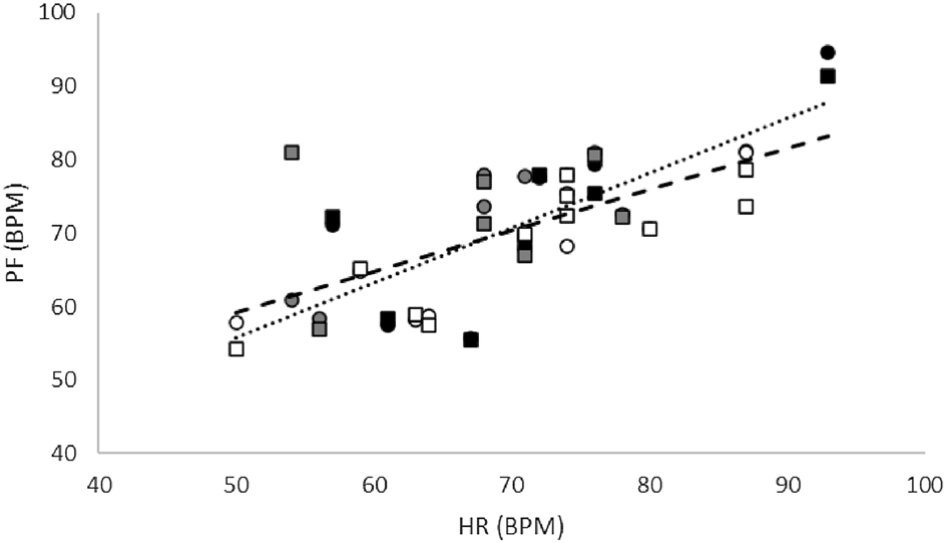
The relationship between pulsation frequency (PF) and heart rate (HR) in units of beats per minute (BPM) in both the retinal vasculature at the optic nerve head (RV; circle data points) and the optic nerve head tissue (ONH_T_; square data points) regions. Black, gray, and white symbol fill colors indicate ND, NDR, and NPDR subjects, respectively. The dotted and dashed lines represent best fit regression lines in RV and ONH_T_ regions, respectively.

**Figure 3:**
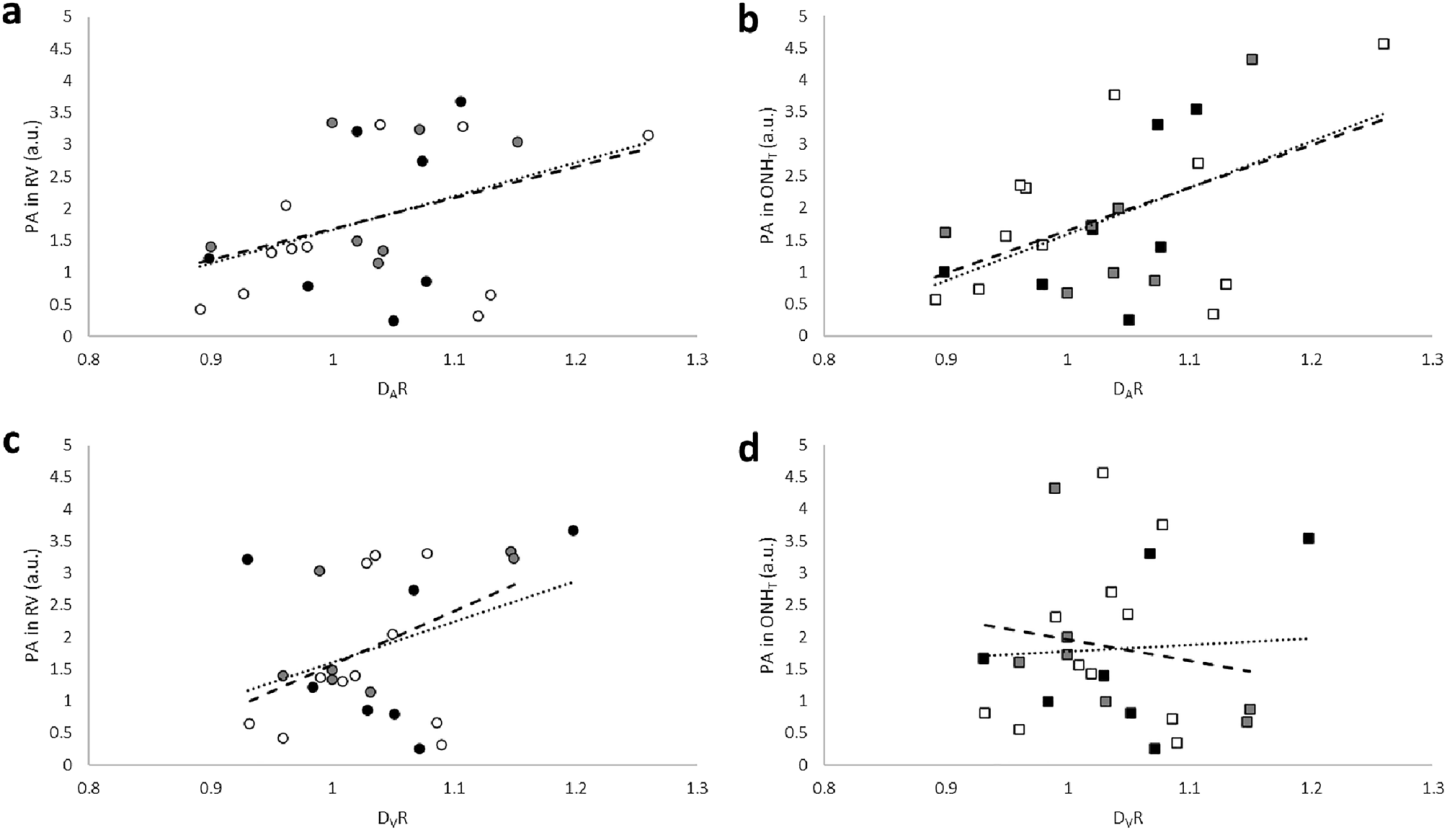
The relationship between pulsation amplitude (PA; arbitrary units, au) and light flicker-induced ratios of arterial diameter (D_A_R) in (**a**) retinal vasculature at the optic nerve head (RV) region and (**b**) optic nerve head tissue (ONH_T_) region. The relationship between PA and light flicker-induced ratios of venous diameter (D_V_R) in (**c**) RV region and (**d**) ONH_T_ region. Black, gray, and white symbol fill colors indicate ND, NDR, and NPDR subjects, respectively. The dotted and dashed lines indicate best fit regression lines including data from all subjects (N = 25) and excluding data from ND subjects (N = 18), respectively.

For both ROIs, there was a significant relationship between PF and HR (r ≥ 0.64, *p* ≤ 0.001), as shown in Fig. 2. There was no significant relationship between PA and IOP for either ROI (*p* ≥ 0.32). The relationships between PA and vasodilatory responses in the RV and ONH_T_ regions are shown in Fig. 3. In the RV region, there was a significant positive relationship between PA and D_A_R (r = 0.53, *p* < 0.01, N = 25). Without data from ND subjects, this relationship tended towards significance (r = 0.43, *p* = 0.08, N = 18). Moreover, there were significant positive relationships between PA and D_V_R with and without data from ND subjects (r ≥ 0.44, *p* ≤ 0.05). In the ONH_T_ region, there were significant positive relationships between PA and D_A_R with and without data from ND subjects (r ≥ 0.50, *p* ≤ 0.03). Moreover, there were no significant relationships between PA and D_V_R with and without data from ND subjects (r ≤ 0.15, *p* ≥ 0.33).

## Discussion

We describe a new retinal imaging system for combined assessment of ONH vascular pulsation and stimulated retinal vasodilation. This system used a new IPPG-derived temporal waveform fitting approach that differentially quantified pulsation metrics (PA and PF) from the retinal vasculature at the ONH (RV) and in the ONH tissue (ONH_T_). In general, PA represents the magnitude of the change in blood volume that occurs during the cardiac cycle, whereas PF represents the frequency of those blood volume changes. In contrast to other methods which evaluate PA in single peripapillary retinal vessels, our method measures PA based on the cumulative response from the retinal vasculature or ONH tissue. Here, we demonstrate the relation of ONH vascular pulsation metrics to stimulated retinal vasodilatory response to light flicker.

### Pulsation metrics

Inter-observer variability of pulsation metrics was low, indicating adequate reproducibility. There was no significant difference in PA or PF between RV and ONH_T_ regions, though periodic waveforms fitted better to the data in the RV region. This is consistent with a higher signal from blood volume changes arising in the large retinal vasculature in contrast to capillaries embedded within the ONH tissue. This difference in the signal strength also accounts for the larger observed inter-image variability of PA in ONH_T_ compared to RV. Nevertheless, pulsation signal was found in both regions and the signal originating from ONH_T_ may provide insight into microvascular pathophysiology of glaucoma^13^ and other retinal vascular diseases.

### Relation of PF to systemic and ocular metrics

As expected, there was a significant relationship between PF and HR. IOP is known to affect retinal vascular pulsation, and a previous study demonstrated that retinal venous PA was reduced with pharmacologically decreased IOP in healthy subjects^4^. We did not observe this relationship in the current study, which is likely due to the small variations in IOP among subjects under ocular normotensive condition. In fact, in an experimental animal model of variable IOP, we did observe a significant relationship between PA and IOP (unpublished data).

### Relation of PA to vasodilation

We report for the first time a relationship between PA and retinal vasodilatory response to light flicker. During light flicker stimulation, retinal vasculature dilates to augment blood flow as part of an autoregulatory response under normal physiological conditions. Previous studies in diabetes have shown reduced retinal vasodilatory response to light flicker^27,31,32^ which is consistent with increased vessel stiffness (or, equivalently, decreased vessel compliance)^33–35^. Decreased venous compliance at the ONH has been shown to manifest in decreased PA^16^. Therefore, altered vessel compliance may be an underlying factor accounting for the relationship between PA and the retinal vasodilatory response to light flicker. In the current study we demonstrated that PA, in both the large retinal vessels and the capillaries of the ONH tissue, was related to D_A_R. However, only in the large retinal vessels was PA related to D_V_R. The lack of correlation between PA in the capillaries of the ONH tissue and D_V_R suggests some decoupling of flow characteristics from artery to vein and restriction of flow within the capillaries. While further study is required, these findings suggest that PA may potentially be an indicator of vasodilatory capacity.

This study had several limitations. First, there was a relatively small number of subjects in each disease group, thus limiting the statistical power to compare metrics among groups. Nevertheless, combining data from all stage groups provided a sufficient range in the data to assess relationships. Future studies with more subjects would be useful. Second, performance of the Hessian-based Frangi filter has been shown to be dependent on the selection of geometric settings which may preclude the segmentation of both large and small vessels within a given image^36^. However, in the current study, the Frangi filter was only used to segment large vasculature at the ONH which are consistent in size. Moreover, vessel geometric and thresholding settings to segment the vasculature with the Frangi filter were manipulated by a trained observer using visual inspection to minimize erroneous segmentations. Indeed, pulsation waveforms were observed in the segmented vasculature, indicating sufficient segmentation performance. Nevertheless, the inclusion of other, secondary algorithms may prove useful to further optimize segmentation. Third, the IPPG signal from the RV region may include some absorption from the ONH_T_. However, given the difference in relationships between metrics depending on the region, the current technique provided enough sensitivity to differentiate regional signals. Fourth, the threshold for goodness of waveform fits used in the exclusion of subjects was based on the data distribution from the current study. Future studies with more subjects allowing for a higher threshold would be useful to better elucidate the phenomena found in the current study. Fifth, systemic metrics (HR and MAP) were acquired immediately prior to imaging, thus asynchronous with the pulsation metrics, in contrast to other studies which continuously measured these variables^4,5^. Variations in systemic metrics over time during imaging may contribute to discrepancy between systemic and ocular metrics. Nevertheless, we observed a highly significant correlation between PF and HR, suggesting the asynchronous measurements between metrics had a minimal effect on the results of the study.

In summary, a new retinal imaging system is described for the correlative assessment of ONH vascular pulsation and stimulated vasodilatory dynamics. The findings of this study suggest pulsation metrics measured at the ONH may serve as biomarkers for vascular compliance and hemodynamics. Future application of the method may be useful to elucidate the contribution and development of vascular factors in retinal diseases.

## Data Availability

The data generated are available from the corresponding author on reasonable request.
